# A 17 Year Old With Developmental Delay Presenting With Increasing Confusion and Imbalance

**DOI:** 10.1002/acn3.70436

**Published:** 2026-06-15

**Authors:** Wei Zhao, Yingli Zhang, Hongliang Zheng

**Affiliations:** ^1^ Neurology Department, Ji'ao Brain Hospital of Siping Siping Jilin Province China; ^2^ Neurology Department, Panjin Central Hospital Panjin Liaoning Province China

**Keywords:** hyperhomocysteinemia, methylmalonic acidemia, tethered cord syndrome

## Abstract

Methylmalonic acidemia is an autosomal recessive genetic disorder primarily caused by defects in methylmalonyl‐CoA mutase and cobalamin (vitamin B12) metabolism. These defects disrupt the tricarboxylic acid cycle and oxidative phosphorylation, leading to the abnormal accumulation of metabolic products such as methylmalonic acid, propionic acid, and methylcitric acid. This accumulation results in damage to multiple systems, including the nervous, hepatic, and renal systems. Tethered cord syndrome results from pathological spinal cord traction due to congenital/acquired factors, causing conus medullaris malposition, neuronal degeneration, and progressive sensorimotor deficits. This report details a diagnostically complex case highlighting an unusual clinical intersection between these two conditions.

This case describes a 17‐year‐old adolescent with a background of developmental delay who presented with progressively worsening confusion and imbalance. The patient's clinical presentation was complex, involving multisystem symptoms. Upon unraveling the complexity, it was confirmed to be an overlap of two distinct disease entities, which led to a broad differential diagnosis and resulted in the patient being repeatedly misdiagnosed and mismanaged. This report details the diagnostic challenges in this complex case, highlighting the rare clinical intersection between these two conditions.

## 1 Part I: History of Presenting Illness (Typically 2–5 Paragraphs, Whole Sentences, Not Bullet Points)

Sixteen years prior, his family noticed signs of developmental delay and gradual cognitive impairment. Specifically, he began walking at 16 months after birth, exhibited slow responses (attention, memory, orientation, and calculation ability have decreased significantly), and an inability to concentrate during primary school (at Age 6). He displayed excessive hyperactivity, poor academic performance, introversion, and reduced verbal communication upon entering junior high school (at Age 12), and eventually dropped out of school after completing junior high school. There was no apparent impact on growth or other motor function. He was diagnosed with “cerebral palsy,” and no specific treatment was provided.

Three months prior to admission, his cognitive impairment worsened, characterized by delayed responses, an inability to answer questions, and a tendency to respond with “I don't know” or “I don't understand”. He also had weakness in both legs and imbalance. His family also noticed inappropriate laughter, delusions of worm contamination in rice, and sleeping with his jackets. He had several episodes of emesis during meals, bowel incontinence, and enuresis. He lost 13 pounds over 1 month.

He was admitted to an outside hospital. MRI brain revealed patchy abnormal T2 hyperintensities adjacent to the posterior horn of the left lateral ventricle. An electroencephalogram showed a reduction in dominant alpha wave frequency and intermittent irregular low‐amplitude slow waves in the bilateral frontal, central, and temporal regions. He was diagnosed with “encephalitis” and treated with methylprednisolone injection (500 mg/day, tapered every 3 days) and intravenous immunoglobulin (2 g/kg over days), without significant improvement in his symptoms. He was discharged on a prednisone taper; however, his symptoms gradually worsened.

He was referred and admitted to our department for a systematic diagnostic workup. At the time of admission, he was inattentive, was unable to walk independently, and could not stand from a squatting position. He also had swelling of the right lower extremity, urinary incontinence (5–6 times), and fecal incontinence (3 times).

## 2 Part II (Whole Sentences or Bullet Points Are Acceptable)

- Neurologic review of symptoms: Cognitive impairment worsened, characterized by delayed responses, an inability to answer questions, and a tendency to respond with “I don't know” or “I don't understand”, weakness in both legs, imbalance.
- General review of systems: Constitutional: Poor appetite resulting in significant weight loss, hair loss with a yellowish tint to hair. Psychiatric: Increased anxiety and fear, abrupt statements (“flew away”, “lost”, “ran away”), inappropriate laughter, delusions of worm contamination in rice, sleeping in outerwear. Gastrointestinal: Emesis during meals, bowel incontinence. Genitourinary: enuresis.
- Past medical/surgical history: Non‐contributory.
- Medications: Unobtainable.
- Social history: Non‐contributory.
- Family history: Non‐contributory.

## 3 Part III: Examination (Videos Are Welcome if Consent has Been Obtained; Whole Sentences or Bullet Points)

- General: Vital Signs: T 36.9°C, P 66/min, R 14/min, BP 120/80 mmHg.
  - General Appearance: Sparse and yellow hair; slight emaciation with reduced subcutaneous fat; pitting edema of the right lower extremity.
  - Cardiovascular: No abnormalities.
  - Respiratory: No abnormalities.
  - Abdominal: No abnormalities.
  - Peripheral Vascular: Weak dorsalis pedis artery pulsation bilaterally.
- Mental status:·Alert but with delayed speech.
  - Impaired attention, memory, orientation, and calculation
  - MMSE score: 3 points; MoCA score: 0 points.
  - Passive eye contact; inappropriate laughter; loss of insight.
- Cranial nerve: Normal.
- Motor:·Strength (MRC grading):
  - Upper limbs: 5/5 throughout.
  - Lower limbs: Proximal 3/5; dorsiflexion/plantarflexion 4/5.
  - Difficulty squatting and standing up.
  - Normal muscle volume and tone in all limbs.
  - Fasciculations: Absent.
- Reflexes:
  - Upper limbs: Tendon reflexes present.
  - Lower limbs: Knee and Achilles tendon reflexes decreased.
  - Pathological Reflexes: Positive bilaterally for Hoffmann, Oppenheim, Gordon, Chaddock, and Babinski.
  - Ankle clonus: Positive bilaterally.
- Sensory: Diminished sensation below L1 for pain, temperature, vibration sensation, and joint position sense.
- Coordination:· Alternating movements: Clumsy in both upper limbs.
  - Finger‐to‐nose test: Poor bilaterally.
  - Distance discrimination: Poor bilaterally.
  - Heel–knee‐tibia test: Inaccurate bilaterally.
- Gait: Spastic.

## 4 Part IV: Diagnostics

- Imaging (please include cut/paste shots of relevant findings—screen shots of MRI or CT findings for example).
  - MRI brain (without contrast) and MRA head (Figure 1):
    ○ Nonspecific patchy T2‐FLAIR hyperintensities in the white matter adjacent to the anterior horn of the left lateral ventricle.
    ○ Mild generalized brain atrophy.
    ○ No intracranial vascular abnormalities.
    **FIGURE 1:**
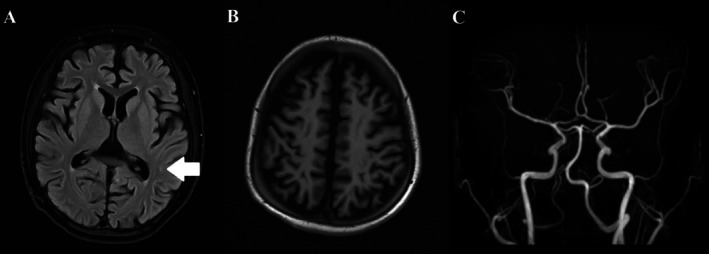
Brain MRI and MRA findings. Image A (T2‐FLAIR): Subcortical T2‐flair hyperintensity. Image B (T1‐weighted): Demonstrates cerebral atrophy. Image C (MRA): No abnormalities observed in the intracranial vasculature.
  - Full spine MRI (Figure 2):
    **FIGURE 2:**
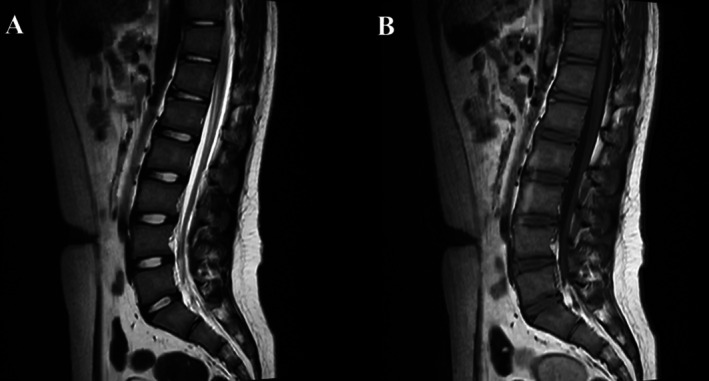
Lumbar spine MRI. Lumbar spine MRI Reveals a low‐lying conus medullaris terminating at the L3 level with a thickened filum terminale (approximately 4 mm in diameter), suggestive of tethered cord syndrome. Image A (T2‐weighted image) and image B (T1‐weighted image) both illustrate these findings.
    Low‐lying conus medullaris terminating at the L3 level with a thickened filum terminale (approximately 4 mm in diameter), suggestive of tethered cord syndrome.
  - Color Doppler ultrasound of both lower extremities:
    ○ No abnormalities in arteries.
    ○ Deep vein thrombosis in the right common femoral vein, middle and lower segments of the femoral superficial vein and great saphenous vein.
  - Chest CT:
    Inflammatory changes in the lower lobes of both lungs, demonstrating patchy high‐density opacities with well‐defined margins.
  - Electrocardiogram, echocardiography, carotid artery ultrasound, and transcranial Doppler ultrasound showed no significant abnormalities.
- CSF Analysis results.
  - Cerebrospinal fluid pressure: 110 mmH2O.
  - RBC count: 0.
  - Protein count: 346.6 mg/L.
  - Glucose: 2.98 mmol/L.
  - Cell count: 0.
  - Cerebrospinal fluid multiplex pathogen targeted detection (tNGS): No bacteria, viruses, fungi, or special pathogens detected.
  - Blood and cerebrospinal fluid autoimmune encephalitis 10 items (NMDAR, AMPAR1, AMPAR2, GABABR, LGI1, CASPR2, IgLON5, DPPX, D2R, GAD65): Negative.
- Serum studies.
  - Blood routine: RBC 3.76 × 10^12/L, HCT 39.4%, MCV 104.7 fL, MCH 35.1 pg.
  - Liver function: Total bile acid 21.8 μmol/L, glycocholic acid 6.78 μg/mL.
  - Homocysteine (HCY): 106 μmol/L.
  - Uric acid: 603 μmol/L.
  - Folic acid: 11.86 nmol/L (reference range: 7.25–44.41 nmol/L).
  - Vitamin B12: 224.1 pmol/L (reference range: 132.84–676.01 pmol/L).
  - Hepatitis, syphilis, and HIV: Negative.
  - Blood tandem mass spectrometry:
    ○ Propionyl carnitine (C3): 8.014 (normal range: 0.5–4).
    ○ Acetylcarnitine (C2): 12.326 (normal range: 6–30).
    ○ C3/C2 ratio: 0.650 (normal range: 0.04–0.25).
- Electrophysiologic studies: Not performed or reported in the original text.
- Other special tests (i.e., pathology with images).
  - Urine organic acid gas chromatography:
    ○ Methylmalonic acid‐2: 196.06 (normal range: 0.20–3.60).
    ○ Methylcitric acid‐4(1): 4.37 (normal range: 0–1.1).
    ○ Methylcitric acid‐4(2): 3.79 (normal range: 0–1).
    ○ Identified two heterozygous pathogenic variants in the MMACHC gene (Figure 3): c.482G > A (p.Arg161Gln, inherited from father) and c.609G > A (p.Trp203Ter, inherited from mother).
      **FIGURE 3:**
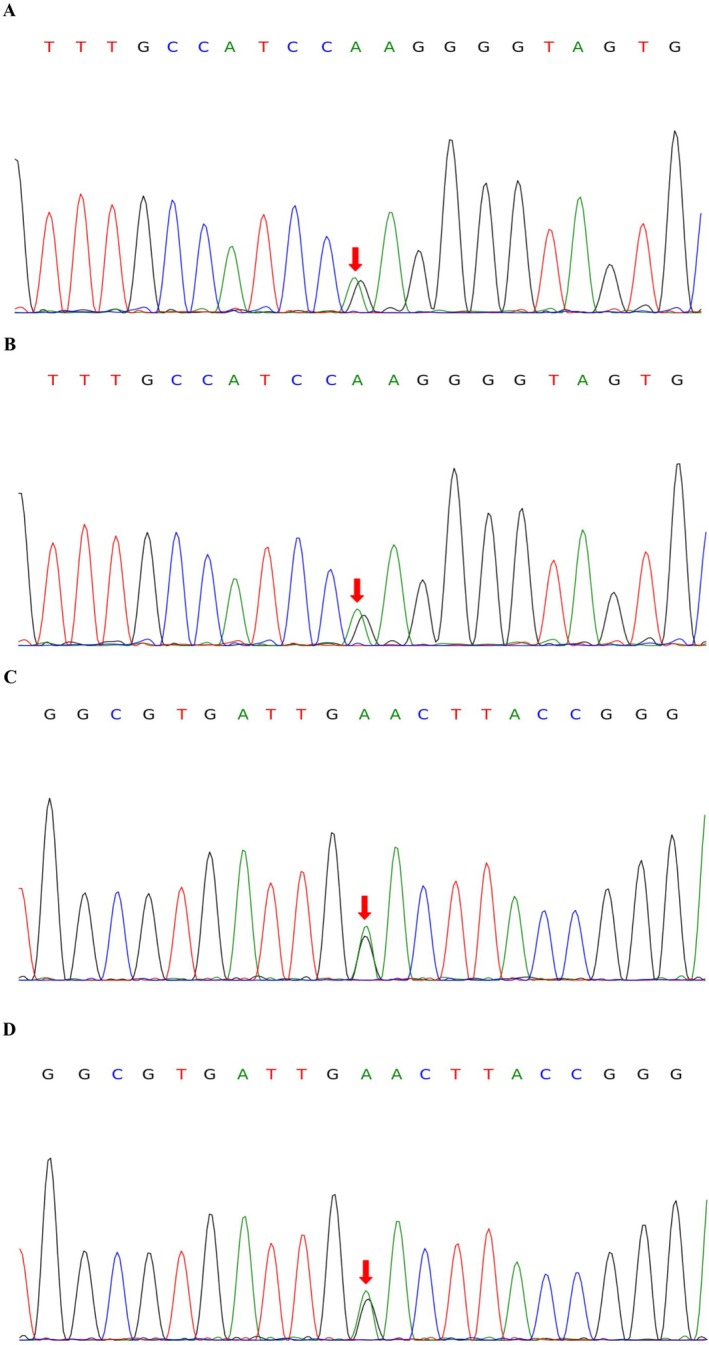
Family pedigree and genetic testing results. Image A: The proband exhibits a heterozygous c.482G>A mutation. Image B: The proband’s father carries a heterozygous c.482G>A mutation. Image C: The proband harbors a heterozygous c.609G>A mutation. Image D: The proband’s mother carries a heterozygous c.609G>A mutation.

## 5 Part V: Diagnosis (1–2 Pages, Full Sentences)

Methylmalonic acidemia (MMA); Tethered cord syndrome (TCS).

## 6 Part VI: Discussion

- What is it: MMA is an inherited organic aciduria caused by pathogenic variants in genes such as MMUT, which lead to a deficiency in the vitamin B12‐dependent mitochondrial enzyme methylmalonyl‐CoA mutase. This enzyme deficiency disrupts the final step in propionyl‐CoA metabolism, preventing the isomerization of L‐methylmalonyl‐CoA to succinyl‐CoA. Consequently, toxic metabolites like methylmalonic acid accumulate in tissues and bodily fluids, causing widespread mitochondrial dysfunction and severe multisystem damage. Neurological manifestations are prominent and can include developmental delay, intellectual disability, seizures, encephalopathy, and structural brain injuries such as basal ganglia lesions and cerebral atrophy.
  The disease is classified into isolated MMA and combined MMA (with elevated homocysteine), with the cblC‐type being the most common form of combined MMA in China.
- How is it diagnosed:
  The diagnosis of MMA is suspected based on clinical presentation and initial laboratory findings. Key diagnostic tests include:
  - Plasma and Urine Metabolite Analysis: Elevated levels of methylmalonic acid and homocysteine are hallmark findings. Supportive laboratory abnormalities can include metabolic acidosis, ketosis, hyperammonemia, and hematologic abnormalities like neutropenia or pancytopenia.
  - Specific Biochemical Testing:
    ○ Blood Tandem Mass Spectrometry (MS/MS): Reveals elevated C3 (propionylcarnitine) levels and an elevated C3/C2 ratio.
    ○ Urine Organic Acid Analysis by Gas Chromatography–Mass Spectrometry (GC–MS): Shows elevated levels of methylmalonic acid and methylcitrate, which are relatively specific biomarkers for MMA.
  - Definitive Diagnosis: Confirmation is achieved through molecular genetic testing to identify the pathogenic variants. In many regions, including the United States, MMA is included in newborn screening panels.
- What is the treatment:
  The treatment for MMA, particularly the cblC‐type, is multifaceted and involves acute and long‐term management:
  - Acute Management: Involves hydration and correction of metabolic abnormalities such as acidosis and hyperammonemia.
  - Long‐Term Management:
    ○ Intramuscular Hydroxocobalamin: Administration of this form of vitamin B12 is a cornerstone of treatment for responsive forms like cblC.
    ○ Dietary Modifications: Protein‐restricted diets, often with specialized medical formulas, are implemented to reduce the intake of precursor amino acids.
    ○ Supplemental Medications: Oral L‐carnitine, betaine, and folic acid are commonly prescribed to aid in detoxification and lower homocysteine levels.
  - Medications to Avoid: Certain drugs, such as valproic acid (which can deplete carnitine) and metformin (which can induce B12 deficiency) should be avoided.
- Any other pearls or interesting aspects of the case or the disease to discuss: This case presents several interesting and instructive aspects:
  1. Phenotypic Variability: MMA can have a wide spectrum of clinical presentations. Beyond the typical neurological and metabolic symptoms, rare manifestations can include skin lesions (cheilitis, dermatitis), intractable diarrhea, and atypical hemolytic uremic syndrome.
  2. Masquerading as Other Conditions: Due to early‐onset developmental delay, patients with MMA are often initially misdiagnosed with cerebral palsy. A rapid clinical decline or the presence of specific laboratory abnormalities (like macrocytic anemia with normal B12 and elevated homocysteine) should prompt a workup for an inborn error of metabolism.
  3. Neuroimaging Spectrum: Neuroimaging findings vary with age of onset. Early‐onset cblC disease is characterized by white matter edema, leukoaraiosis, and basal ganglia lesions. Later‐onset cases more commonly show cerebral atrophy and multifocal white matter abnormalities.
  4. Unique Co‐occurrence with Tethered Cord Syndrome (TCS): This case is unique due to the co‐occurrence of MMA and TCS. While MMA can cause spinal cord pathology resembling subacute combined degeneration (SCD), the patient's symptoms (lower limb weakness, sensory level, urinary/fecal incontinence) were consistent with conus medullaris and cauda equina syndrome from TCS, not classic SCD. This combination made the case prone to misdiagnosis and complicated the clinical picture, as the spinal symptoms did not improve with metabolic treatment.

## 7 Part VII: Outcome (1–3 Paragraphs Describing the Clinical Outcome, Full Sentences)

After 14 days of intensive inpatient treatment targeting the metabolic disorder, the patient showed significant improvement in several areas. His appetite and mental status improved notably; his speech became more coherent, and he was able to engage in simple conversations, answer questions, and recognize his parents. Metabolic parameters also responded favorably to therapy.

However, the neurological deficits related to his spinal cord condition did not resolve. The weakness in his lower limbs persisted, and he remained unable to walk independently. Upon discharge, he was referred to neurosurgery for evaluation of TCS.

At a five‐month follow‐up, the patient continued to show positive metabolic outcomes. His hair color had normalized, and he could communicate normally. He achieved ambulation with the assistance of a walker. Laboratory studies showed a significant reduction in homocysteine levels (58 umol/L) and a normal D‐dimer. Despite these improvements, the family declined the recommended surgical intervention for TCS. The patient was managed conservatively, adhering to the prescribed metabolic regimen. The final diagnosis remained cblC‐type Methylmalonic Acidemia combined with Tethered Cord Syndrome.

## 8 Part VIII: Take Home Points—What Should They Have Learned From This Case (Minimum of Three Bullet Points)

- When encountering patients with developmental delay, cognitive dysfunction, signs and symptoms of SCD, and abnormal laboratory levels, the possibility of MMA should be considered.
- The diagnosis of MMA mainly relies on genetic testing, blood tandem mass spectrometry, and gas chromatography of organic acids in urine.
- The uniqueness of this case of MMA lies in the coexistence of TCS, which makes it extremely prone to misdiagnosis.

## 9 Optional/Suggested

Please submit 1–3 multiple choice questions relevant to any aspect of the case which can be inserted along the way. These can be questions about localization, diagnostic testing decisions, diagnosis and differential, etc.

1. A child with a history of global developmental delay presents with acute encephalopathy, vomiting, and metabolic acidosis. Laboratory studies reveal macrocytic anemia with a normal serum B12 level. Which of the following initial diagnostic tests is most likely to reveal the underlying etiology?
  - Serum Lactate
  - Plasma Homocysteine level
  - EEG
  - Chromosomal Microarray

Answer: B. The combination of neurodevelopmental issues, acute metabolic decompensation, and macrocytic anemia with a normal B12 level is highly suggestive of a disorder like cblC‐type MMA, where homocysteine is elevated. This is a more targeted first test than the other options in this clinical context.

2 A patient with genetically confirmed cblC‐type Methylmalonic Acidemia is started on metabolic treatment with hydroxocobalamin, carnitine, and betaine. Which of the following medications should be avoided in this patient due to its potential to exacerbate the underlying metabolic defect?
  - Levetiracetam
  - Valproic Acid
  - Acetaminophen
  - Ibuprofen

Answer: B. Valproic acid can cause secondary carnitine deficiency, which is particularly dangerous in patients with MMA who often already have low carnitine stores and rely on carnitine for detoxification.

3 The neuroimaging findings in early‐onset cblC disease most characteristically include which of the following?
  - Cerebral aneurysms and subarachnoid hemorrhage
  - Symmetrical basal ganglia lesions and diffuse white matter edema
  - Cortical tubers and subependymal nodules
  - Parietal and occipital cortical infarctions

Answer: B. Symmetrical abnormalities of the basal ganglia and diffuse white matter changes are classic neuroimaging features of early‐onset cblC disease, reflecting the metabolic vulnerability of these structures.

## Author Contributions

**Wei Zhao:** first author, conceived the study, performed experiments, collected data and drafted the original manuscript. **Yingli Zhang:** corresponding author, supervised the research, revised the manuscript critically and approved the final submission. **Hongliang Zheng:** co‐author, assisted with data analysis and literature collation.

## Funding

The authors have nothing to report.

## Conflicts of Interest

The authors declare no conflicts of interest.

## Data Availability

Data sharing not applicable to this article as no datasets were generated or analysed during the current study.

